# Impact of Local Drug Delivery of Minocycline on the Subgingival Microbiota during Supportive Periodontal Therapy: A Randomized Controlled Pilot Study

**DOI:** 10.3390/dj8040123

**Published:** 2020-10-27

**Authors:** Haruna Miyazawa, Takako Nakajima, Makoto Horimizu, Kazuhiro Okuda, Noriko Sugita, Kyoko Yamazaki, Lu Li, Yoshiko Hayashi-Okada, Takuya Arita, Misa Nishimoto, Mieko Nishida, Robert J. Genco, Kazuhisa Yamazaki

**Affiliations:** 1 Research Unit for Oral-Systemic Connection, Division of Oral Science for Health Promotion, Niigata University Graduate School of Medical and Dental Sciences, 2-5274, Gakkocho-dori, Chuo-ku, Niigata 951-8514, Japan; haruna-m@dent.niigata-u.ac.jp (H.M.); ndc@nakajima-d-clinic.com (T.N.); k-yamazaki@dent.niigata-u.ac.jp (K.Y.); 2 Division of Periodontology, Department of Oral Biological Science, Niigata University Graduate School of Medical and Dental Sciences, 2-5274, Gakkocho-dori, Chuo-ku, Niigata 951-8514, Japan; horimizum@gmail.com (M.H.); okuda@dent.niigata-u.ac.jp (K.O.); psugita@dent.niigata-u.ac.jp (N.S.); 3 Department of Computer Science and Engineering, State University of New York at Buffalo, 338 Davis Hall, Buffalo, NY 14214, USA; lli59@buffalo.edu; 4 Sunstar Inc., 3-1, Asahimachi, Takatsuki-shi, Osaka 569-1134, Japan; yoshiko.okada@jp.sunstar.com (Y.H.-O.); takuya.arita@jp.sunstar.com (T.A.); misa.nishimoto@jp.sunstar.com (M.N.); 5 Departments of Oral Biology, and Microbiology and Immunology, and Center for Microbiome Research, University at Buffalo, 135 Foster Hall, 3435 Main Street, Buffalo, NY 14214, USA

**Keywords:** supportive periodontal therapy, local drug delivery, minocycline, subgingival microbiota, 16S rDNA

## Abstract

The aim of this study was to examine the effect of adjunct local minocycline administration on the microbiological parameters of subgingival plaque samples in the residual periodontal pockets. Ten chronic periodontitis patients under a supportive periodontal therapy regimen were recruited. After subgingival debridement, either 2% minocycline gel, Periocline™, (Test Group) or a placebo (Control Group) was administered to the selected sites once a week for three weeks. Subgingival plaque was collected at baseline, and at four weeks and eight weeks. The microbiological composition was analyzed by 16S ribosomal RNA sequencing. In the Test Group, α-diversity (evenness) decreased compared to the baseline (*p* = 0.005) and was lower compared to the control group at four weeks (*p* = 0.003). The microbial community composition between the two groups was significantly different at four weeks (*p* = 0.029). These changes were attributable to a decrease in the bacteria associated with periodontitis and an increase in the bacteria associated with periodontal health. Additionally, the improvement in bleeding on probing continued at eight weeks; however, there were little microbial effects of 2% minocycline gel observed at eight weeks. The control group demonstrated no change throughout the eight-week experimental period. Thus, local minocycline administration can change the subgingival microbial community of residual periodontal pockets.

## 1. Introduction

Periodontitis is caused by a complex of pathogenic microorganisms and inflammatory host responses resulting in the destruction of connective tissue attachment and alveolar bone resorption [1]. Although the etiological role of subgingival bacteria is clear, the understanding of the roles of specific bacteria, particularly red complex bacteria (*Porphyromonas gingivalis*, *Treponema denticola* and *Tannerella forsythia*), in the pathogenesis of periodontitis is changing [2]. Among red complex bacteria, the pathogenic role of *P. gingivalis* is proposed to be key to the induction of dysbiosis in the subgingival microbiome [3].

Successful periodontal treatment results in not only the reduction of periodontal pocket depth but also a reduction in the proportion of pathogenic bacteria, such as red complex bacteria, in periodontal pockets [4,5]. Mechanical debridement of bacterial deposits on root surfaces is fundamental to the treatment of periodontitis. However, mechanical debridement is a highly demanding procedure with limitations, such as the inability to access deposits in deep pockets, surface irregularities and furcation areas. In addition, mechanical debridement has limited access to bacteria within the gingiva. These challenges cannot even be solved if access flaps are created. As a result, although the depth of periodontal pockets significantly decreases after scaling and root planing, there may be residual periodontal pockets that require supportive periodontal treatment.

The supportive periodontal therapy (SPT) regimen includes professional periodic mechanical tooth cleaning, a procedure to remove dental biofilm from supra- and subgingival tooth surfaces using ultrasonic and hand instrumentation. Although subgingival microbial aggregates can be removed with mechanical debridement, bacteria start to recolonize soon after treatment [6]. Therefore, despite receiving supportive periodontal therapy, the exacerbation or recurrence of periodontal destruction may take place in a subset of patients, likely associated with residual pocket depths and an incomplete removal of subgingival flora.

The use of antimicrobials is recognized as an adjunctive therapy to mechanical debridement to further reduce pocket depths, especially in patients who have severe or aggressive periodontitis [7,8]. The efficacy of a microcapsule gel containing 2% minocycline HCl (Periocline™, Sunstar Inc., Osaka, Japan) as a local drug delivery system (minocycline belongs to the tetracycline group of antibiotics) has been established as an adjunctive therapy to scaling and root planing in the treatment of periodontitis [9,10]. However, there is little research to show how 2% minocycline gel affects the microbiota including cultivable and uncultivable bacteria residing in the subgingival plaque.

In order to maintain treated periodontal pockets in a stable state, it is important not only to suppress periodontopathic bacteria but also to resolve the dysbiosis in the periodontal pockets, restoring the flora to one that is compatible with health. In order to understand the longitudinal clinical effect of 2% minocycline gel as an adjunct treatment to mechanical debridement, it is worthwhile to understand the effect of 2% minocycline gel on the subgingival microbiome. The purpose of this pilot study is to assess the status and changes in the subgingival microbiota in participants receiving 2% minocycline gel versus a placebo gel as adjunct therapy to ultrasonic scaling during and after supportive periodontal therapy.

## 2. Materials and Methods 

### 2.1. Study Design

This study was approved by the human subjects ethics board of Niigata University Medical and Dental Hospital in Japan (approval number 27-R37-2-10, 29 March 2016) and was conducted in accordance with the Helsinki Declaration of 1975, as revised in 2013. This was a parallel arm of a randomized controlled trial of outpatients from the Departments of Periodontics and General Dentistry at Niigata University Medical and Dental Hospital. All patients provided written informed consent before any study activities took place. 

This study was registered at UMIN Clinical Trials Registry (UMIN000036010) on 26 September 2019; Retrospectively registered, https://upload.umin.ac.jp/cgi-open-bin/ctr_e/ctr_view.cgi?recptno=R000040981.

All patients had been previously treated for chronic periodontitis and then received a supportive periodontal therapy regimen for at least three months. Inclusion criteria consisted of: (1) 35 years of age or older, regardless of gender; (2) good supragingival plaque control; (3) good general health without any remarkable past history of disease except for well-controlled hypertension, dyslipidemia and diabetes; (4) presence of at least 14 remaining teeth; and (5) presence of at least two teeth with probing pocket depths > 5 mm with concomitant bleeding on probing. The exclusion criteria were: (1) use of antibiotics in the past six months; (2) receiving surgical periodontal treatment or scaling and root planing within the past three months; (3) taking steroidal and nonsteroidal anti-inflammatory agents on a daily basis; (4) being a smoker; and (5) having a history of hypersensitivity to tetracycline agents.

Subjects were randomly assigned to one of two groups, Test or Control, using a random-number table. The random-number table was kept by a researcher not directly involved in the study. Subjects and clinicians were blinded to the allocation to the Test or Control group. The test group received 2% minocycline gel (Periocline™); the control group received a placebo gel.

### 2.2. Clinical Examination and Microbiological Sampling

**Figure 1:**
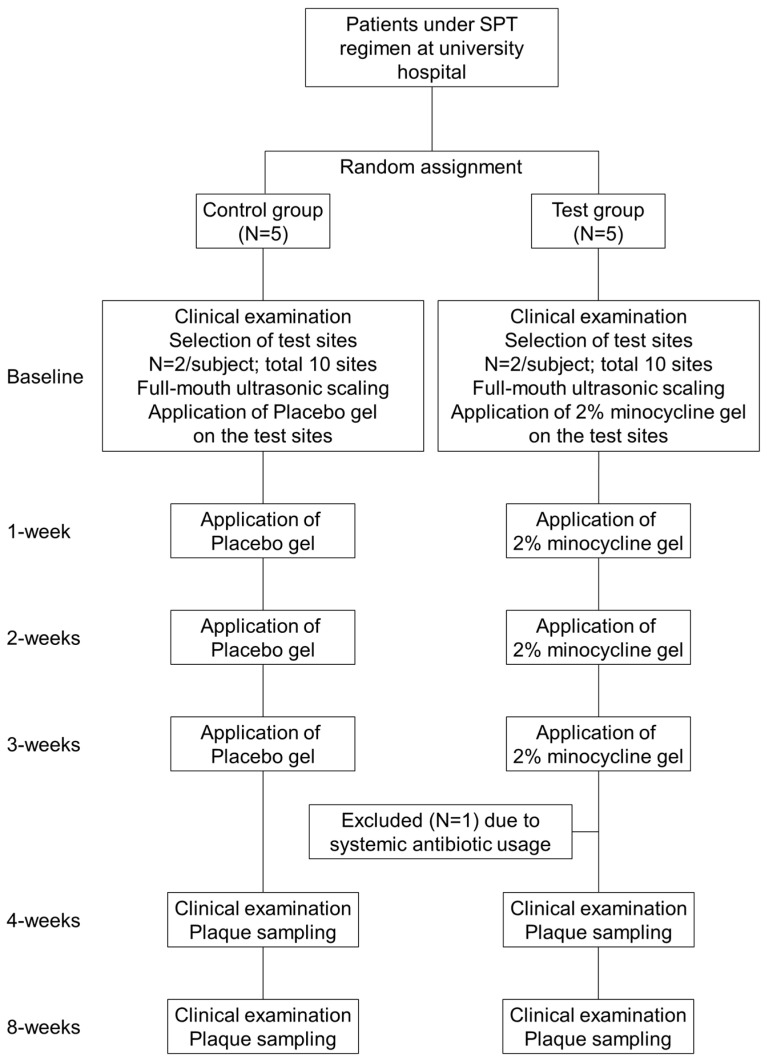
Flow chart of the study.

Figure 1 shows the flowchart of this clinical study. Participants received clinical examinations with periodontal measurements at baseline, and at four and eight weeks after baseline. All examiners were trained and calibrated before the study began. At the baseline visit, supra and subgingival plaque was removed using ultrasonic scalers. After the cleaning, participants were randomized to either the test or control, and the test or placebo gel was administered to the target sites. Target sites were those with probing pocket depth (PPD) > 5 mm and concomitant bleeding on probing. Treatment was repeated once a week for three weeks [11]. 

Periodontal parameters such as (PPD), Bleeding on probing (BOP) and Clinical attachment level (CAL) were measured at six sites per tooth. A plaque control record (PCR, a plaque index) was measured at four sites per tooth at baseline and four and eight weeks after baseline [12]. Sterile Gracey curettes (Hu-Friedy Mfg. Co., Chicago, IL, USA) were used to collect the plaque samples from teeth with PPD ≥ 5 mm and concomitant bleeding on probing; subgingival plaque samples were collected and immediately placed in separate microcentrifuge tubes containing DNase-free TE buffer.

### 2.3. Microbiological Assessment of Subgingival Plaque Samples

Genomic DNA was extracted from plaque samples using the FastDNA SPIN Kit with the FastPrep instrument according to the manufacturer’s instruction (MP Biomedicals, Santa Ana, CA, USA). The counts of total bacteria, *P. gingivalis*, *T. forsythia* and *T. denticola* were determined using a real-time polymerase chain reaction (qPCR, Life Technologies, Tokyo, Japan) with universal and specific primers [13,14,15,16]. The samples were analyzed in duplicate in 20 µL containing 2 µL of template DNA, 10 µL of SYBR Green Master Mix and each specific forward/reverse primer (final concentration of 0.25 µL). The cycling parameters were as follows: 50 °C for 2 min, 95 °C for 10 min, followed by 40 cycles at 95 °C for 15 s and 60 °C for 1 min with a dissociation stage. The number of bacteria in the sample was calculated according to a calibration curve for each specific bacterium.

Metagenomic amplification of the extracted DNA for 16S sequencing of the V3–V4 region proceeded following the Illumina manufacturer protocol (Illumina Inc., San Diego, CA, USA) with modifications [17]. Paired-end sequencing, 2 × 300 bp, was performed using the Illumina MiSeq System (Illumina Inc., San Diego, CA, USA). Operational Taxonomic Units (OTUs) were identified with a >97% sequencing identity using the Quantitative Insights Into Microbial Ecology (QIIME) pipeline and the Human Oral Microbiome (HOMD) database.

### 2.4. Statistical Analysis

Data management and statistical analyses were performed using the SPSS 26 version software (IBM Corp., Armonk, NY, USA) and the R 4.0.0 version software (R Foundation Inc., Vienna, Austria). Clinical and demographic variables were analyzed using the Fisher’s exact test on categorical variables and the Student’s *t*-test or Mann–Whitney U test on continuous variables for a comparison between groups, and the Friedman test followed by Wilcoxon signed-rank test on continuous variables or the Cochran’s Q test followed by McNemar’s test on categorical variables was performed for an intragroup comparison. A Bonferroni correction was used for the multiple-testing correction. The levels of bacteria, such as *P. gingivalis*, *T. forsythia* and *T. denticola,* were analyzed using the Mann–Whitney U test for a comparison between groups and the Friedman test followed by Wilcoxon signed-rank test with a Bonferroni correction for an intragroup comparison. The subgingival microbial α-diversity based on the number of OTUs and the Shannon Index were tested using t-tests for comparisons between groups. For intragroup comparisons, a one-way ANOVA followed by the Dunn–Sidák approach was used. The PCoA plot using weighted UniFrac distance was analyzed. The significance of community structure differences was confirmed using the one-way permutational multivariate analysis of variance (PERMANOVA) test. A probability value (*p*) < 0.05 was considered statistically significant.

## 3. Results

### 3.1. Patients

Subject recruitment started in March 2016 and was completed by the end of June 2016. Table 1 shows the clinical and demographic characteristics of participants. Before the four-week examination, one patient from the test group was excluded because of systemic antimicrobial usage; therefore, four patients (three females and one male) comprised the test group, and five patients (four females and one male) were in the control group; all had complete clinical data. Groups were similar in age, gender distribution, oral hygiene status and teeth sampled. There were no adverse reactions to 2% minocycline gel (Periocline™) or placebo administrations at any time.

### 3.2. Periodontal Parameters

Table 2 shows changes in the periodontal parameters from baseline to four weeks and from the baseline to eight weeks. The periodontal parameters on the targets sites where the test or placebo gel was administered were used for the statistical analysis. There was no difference in the periodontal parameters between the test and control groups at baseline. In Table 2, both groups showed no significant changes in the probing pocket depth (PPD) from the baseline to four or eight weeks; however, the clinical attachment level (CAL) was significantly lower in the test group when compared with the control group at four or eight weeks (*p* = 0.027 and *p* = 0.043, respectively). In the test group, there was a significant improvement in bleeding on probing (BOP) at four and eight weeks when compared with the baseline (*p* = 0.002) and also when compared with the control group (*p* = 0.003 and *p* = 0.015, respectively). Within the control group, there was no significant improvement in BOP from the baseline to four or eight weeks.

### 3.3. Microbiological Parameters

#### 3.3.1. Quantitative Bacteria

The DNA amount in the samples was under the detection limit in two sample at four weeks and in one sample at eight weeks. Therefore, these samples were excluded from the analyses. There was no difference in the average number of 16S rRNA gene copies (which gives an estimate of the number of bacteria in the sample) between the test and control groups at baseline, as shown in Table 3. During the time between the baseline and four weeks, there was no change in the number of bacteria in either group, as assessed by qPCR. However, at four weeks, there was a significant decrease of *P. gingivalis*, *T. forsythia* and *T. denticola* in the test group when compared with the control group (*p* = 0.043, *p* = 0.043 and *p* = 0.006, respectively). The reduction of T. forsythia remained at the eight-week visit, as shown in Table 3.

#### 3.3.2. Diversity

**Figure 2:**
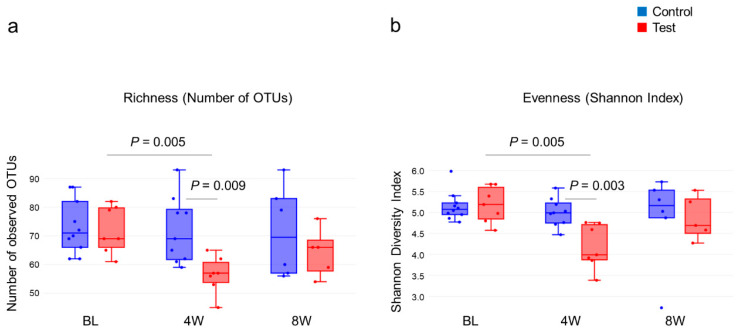
(**a**) Subgingival microbial alpha-diversity based on richness and the number of OTUs, and (**b**) Evenness and the Shannon Index of the genera. For comparison between groups, the parametric *t*-test was used, and the *p*-value was calculated using Monte Carlo permutations. For intragroup comparisons, the *p*-value was determined using one-way ANOVA followed by the Dunn–Sidák approach.

**Figure 3:**
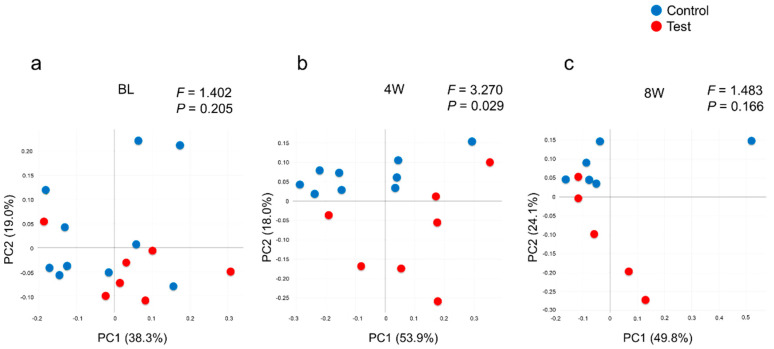
Comparison of subgingival microbial community compositions based on a weighted UniFrac analysis at (**a**) baseline, (**b**) four weeks and (**c**) eight weeks. Plots represent the samples in the control group (blue) and the test group (red). The percent of variance, explained by each principal coordinate (PC), is indicated on the axis. F and *p*-values were obtained using the PERMANOVA test.

From the 16S metagenomic analysis of the samples, 17 baseline samples (10 from the control group, seven from the test group), sixteen four-week samples (nine from the control group, seven from the test group) and eleven eight-week samples (six from the control group, five from the test group) were used. There was no difference in microbial diversity at the genus level (α-diversity, number of OTUs and Shannon Index) between the test and control groups at baseline (Figure 2). The number of OTUs in the test group decreased at four weeks when compared to the baseline, *p* = 0.004, but was not statistically different from the baseline at eight weeks. The control group showed little change in α-diversity throughout. The Shannon Index showed a similar pattern in the number of OTUs, with a reduction at four weeks in the test group and no changes in the control group (Figure 2b). Similar patterns were observed at the species level (data not shown). From Figure 3 it can be seen that weighted UniFrac distances demonstrated no significant difference between the test and control groups at baseline; however, significant qualitative differences in the subgingival microbiota composition were observed at four weeks between the two groups (*p* = 0.029) but were not seen at eight weeks.

#### 3.3.3. Heatmap Analysis

**Figure 4:**
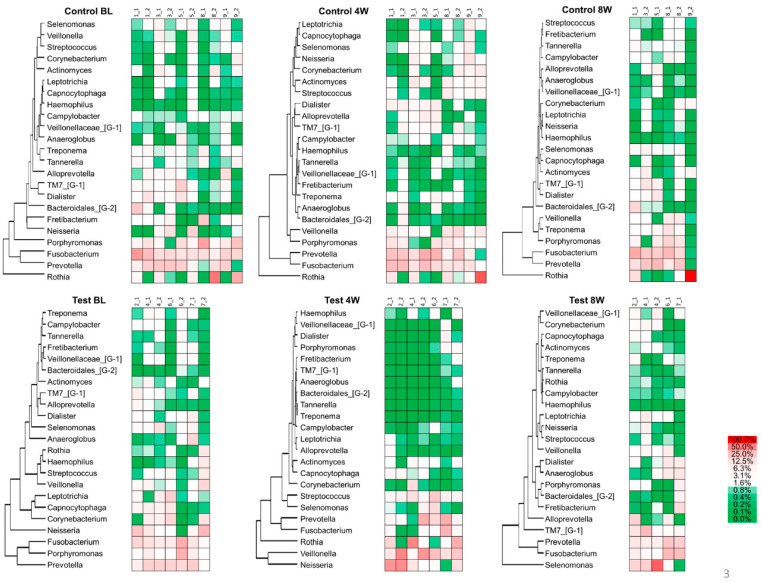
The heat map of relative abundances at the genus level.

The heat map in Figure 4 shows the hierarchical clustering of the subgingival plaque microbiota. Out of 152 genera, the major bacterial genera with a mean relative abundance of >1% were selected for analysis. *Porphyromonas*, *Prevotella* and *Fusobacterium* were overrepresented and associated with each other at baseline in the test and control groups, and throughout the experimental period in the control group. At four weeks in the test group, however, *Porphyromonas* became a minor portion of the population, and its association with *Prevotella* and *Fusobacterium* was reduced. The residential bacteria, such as *Rothia*, *Veillonella* and *Neisseria,* increased. By eight weeks, the associations returned to values close to what they were at baseline.

## 4. Discussion

In this pilot study, the primary finding was qualitative changes in the subgingival plaque microbiome at four weeks after treatment with 2% minocycline gel versus placebo gel. The effect of 2% minocycline gel was examined in periodontal pockets ≥ 5 mm with concomitant bleeding on probing. Prior to recruitment into this study, participants had been placed on a supportive periodontal therapy regimen at three-month intervals. It is common practice to assess the outcome of therapy after three months as it appears that limited improvements occur thereafter [18]. It has been shown that residual periodontal pockets of ≥5 mm are predictive of further attachment and tooth loss [19,20]. In addition, residual periodontal pockets with BOP as an indicator of continued inflammation have been found to be more likely to show deterioration in the future [21].

Recolonization of bacteria in residual periodontal pockets is inevitable during post-SPT periods. It is reported that the initial results obtained following active periodontal therapy could not be sustained using standardized SPT over three years [22]. An increase in the pocket probing depth and a loss of attachment over time, as well as a loss of teeth, was also reported. Consistent with these results, a subgingival debridement with an ultrasonic scaler alone demonstrated only a marginal effect on clinical parameters. Based on these findings, antimicrobials have been used to improve clinical outcomes during and after SPT. The same pattern described in the literature was repeated in this study. Perhaps, periodontal treatment with 2% minocycline gel for longer than four weeks would have helped keep the red complex bacteria at a lower level for a longer time, allowing tissue healing and increasing the ability of the individual to ward off recolonization for longer than four weeks. A longer, larger clinical trial is needed to answer this question; additionally, an analysis of inflammatory markers, perhaps in gingival crevicular fluids, corresponding to changes in microbiota may be helpful to better understand the response to periodontal treatments.

The main purpose of the present study was to examine the effect of adjunctive 2% minocycline gel administration with SPT on microbiological parameters. Without antimicrobials, no effect of subgingival debridement was observed, not only for microbiological but also for clinical parameters. It is known that there is a significant decrease in the number of periodontal pathogens immediately after debridement [23,24]. Some studies found that over time, usually three to six months, the microbiota appear to regrow to the same load or composition as before subgingival debridement [25,26], and others found little effect on the bacterial load [27,28]. The reasons for the differing results are not known.

In contrast to the placebo, the 2% minocycline gel administration resulted in a significant reduction of red complex bacteria, the percentage of BOP-positive sites and CAL, despite there being no significant difference for PPD or the total number of bacteria. The suppressive effect on *T. forsythia* persisted until eight weeks after the baseline, which was four weeks after the last administration of 2% minocycline gel. The observed clinical effect is difficult to compare with other studies. Whereas there are a number of studies showing the additional clinical effects of locally administered antimicrobials with initial scaling and root planing, there is a paucity of research for the adjunctive use of antimicrobials after ultrasonic SPT. One study evaluated the efficacy of slow-release doxycycline gel administered with nonsurgical therapy in subjects with recurrent or persistent periodontitis. The authors concluded that the therapy may provide a short-term benefit in controlling inflammation and deep pockets in treated periodontal patients [29]; however, this study did not assess the microbiological status.

The most remarkable point of this study is the effect of the 2% minocycline gel on the subgingival plaque microbiota at four weeks in the test group. In total, 539 OTUs were identified. At baseline, there was no difference in the numbers of organisms between the test and control groups. However, after four weeks of 2% minocycline gel treatment, there was a significant difference in the community diversity with significantly lower numbers of OTUs identified in the test group versus the control group (Figure 2a). This finding is consistent with other studies [30,31], with one exception [32]. It is known that sites with severe disease harbor a more complex bacterial community than the one seen with slight/moderate disease. Drastic changes in the redox environment and nutritional conditions in deep periodontal pockets allow the growth of specific bacteria, allowing periodontal disease to flourish. Therefore, with periodontal disease, a higher oral community diversity is associated with disease. This finding is in contrast with what is seen with the gut microbiota, where a greater diversity is associated with health and a low diversity with disease [33,34].

After the 2% minocycline gel administration, a decreased number of disease-associated bacteria significantly affected the overall community composition (Figure 2b and Figure 3). However, these changes disappeared by eight weeks, suggesting a limited duration of effect. As shown in Figure 3, the composition of microbiota in the subgingival samples was different from site to site, even in the same patient. However, *Porphyromonas*, *Prevotella* and *Fusobacterium* were consistently abundant genera across the patients at baseline. In contrast, the abundance of emerging periodontitis-associated bacteria [35,36] such as *Filifactor* and *TM7* varied from participant to participant (Figure 4 and Supplementary Figure S1). In the present study, varying amounts of these emerging periodontitis-associated bacteria were detected in the sites. Whereas *Fretibacterium*, *Filifactor* and *TM7* were suppressed by 2% minocycline gel in the test group at four weeks, the continued effect of mechanical debridement was found only for *Fretibacterium*. 

*F. alocis*, a member of the genus *Filifactor*, is not only present at the site of periodontitis but has also been shown to induce the production of inflammatory cytokines such as TNF-α, IL-1β and IL-6 in vitro [37,38]. In addition, many *Fretibacterium*, including *F. fastidiosum* and *TM7* (the TM7 phylum was named ‘Candidatus Saccharibacteria’ based on the complete genome sequence), are still difficult to culture. It has been reported that *TM7x* was cultured successfully by parasitizing *Actinomyces odontolyticus* [39], but its function is still unclear. In the future, advances in biome analysis and cultivation techniques are expected to elucidate the effects on the host and the relationship among microorganisms. 

This is the first study to report the effect of the local administration of 2% minocycline gel on emerging periodontitis-associated bacteria. In addition, it is noteworthy that at four weeks the 2% minocycline application induced an increase in the proportion of *Rothia*, *Veillonella* and *Neisseria*, possible periodontal health-associated bacteria, suggesting an improvement in the periodontal microbiome.

## 5. Conclusions

In this pilot study, we demonstrated that the local administration of 2% minocycline gel, Periocline™, a slow-release formulation of minocycline [40], was clearly effective in decreasing not only red complex bacteria but also emerging periodontitis-associated bacteria. 2% minocycline gel use was also associated with an increase in health-associated bacteria. These results are needed in periodontal disease treatment. The duration of the effect was at least four weeks but was not maintained during the usual/historical SPT intervals of eight weeks or longer. Although the bacterial composition returned to a dysbiotic state eight weeks after the baseline in the 2% minocycline treated group, the percent of sites showing BOP remained low at eight weeks, suggesting that the bacterial composition was not inflammophilic and could be different from that at baseline. Although the limitations of this study are that the numbers of participants are small, which can lead to a low statistical power, and the study had a short duration (eight weeks), the findings are new and could be important in future treatment plans.

## Figures and Tables

**Table 1:** Clinical and demographic features of participants.

Mean ± SD	Control (*n* = 5)	Test (*n* = 4)	*p*-Value
Age (years)	67.4 ± 11.1	65.5 ± 2.1	0.72
Gender, female (%)	80	75	1.00
Whole-mouth PPD (mm)	2.41 ± 0.30	2.13 ± 0.36	0.32
Whole-mouth CAL (mm)	3.05 ± 0.57	2.88 ± 0.29	0.63
Whole-mouth BOP (%)	5.36 ± 3.43	5.09 ± 3.49	0.92
Whole-mouth PCR (%)	3.75 ± 2.93	14.39 ± 16.35	0.34

Abbreviations: PPD, probing pocket depth; CAL, clinical attachment level; BOP, bleeding on probing; PCR, plaque control record. *p*-value was determined by a Student’s *t*-test for Age, PPD, CAL, BOP, PCR and by a Fisher’s exact test for Gender.

**Table 2:** Changes in the periodontal parameters of the test sites from the baseline to four weeks and eight weeks.

		Control (*n* = 10)	Test (*n* = 8)	*p*-Value ^1^
PPD (mm)	BL	6.20 ± 1.32	5.50 ± 0.53	0.315
mean ± sd	4W	6.20 ± 1.32	5.38 ± 0.74	0.237
	8W	6.20 ± 1.14	5.13 ± 0.99	0.101
	*p*-value ^2^	1.000	0.607	
CAL (mm)	BL	6.90 ± 1.37	5.88 ± 0.83	0.122
mean ± sd	4W	6.70 ± 1.25	5.38 ± 0.74	0.027
	8W	6.80 ± 1.23	5.63 ± 0.74	0.043
	*p*-value	0.549	0.171	
BOP (positive %)	BL	100	100	1.000
	4W	90	13 **	0.003
	8W	80	13 **	0.015
	*p*-value	0.368	0.002	
PCR (positive %)	BL	20	25	1.000
	4W	20	13	1.000
	8W	10	13	1.000
	*p*-value	0.717	0.717	

^1^*p*-value between test and control; ^2^*p*-value within test or control. ** *p* < 0.05 in comparison with baseline. Abbreviations: PPD, probing pocket depth; CAL, clinical attachment level; BOP, bleeding on probing; PCR, plaque control record. PPD and CAL: the Mann–Whitney U test was used for a comparison between groups, and the Friedman test followed by the Wilcoxon signed-rank test with a Bonferroni correction was used for an intragroup comparison. PCR and BOP: the Fisher’s exact test was used for a comparison between groups, and the Cochran’s Q test followed by the McNemar’s test with a Bonferroni correction was used for an intragroup comparison.

**Table 3:** Changes in the total amount (log) of bacteria, and in *Porphyromonas gingivalis*, *Tannerella forsythia* and *Treponema denticola*.

		Control	Test	*p*-Value ^1^
Total bacteria	BL	6.30 ± 1.00	6.85 ± 0.72	0.408
	4W	5.67 ± 1.92	4.97 ± 2.44	0.515
	8W	5.44 ± 2.14	5.12 ± 2.83	0.897
	*p*-value ^2^	0.836	0.607	
*Porphyromonas gingivalis*	BL	3.80 ± 1.67	3.97 ± 1.98	0.696
	4W	3.36 ± 1.47	1.73 ± 1.57 *	0.043
	8W	2.50 ± 1.81	1.77 ± 1.64	0.360
	*p*-value	0.117	0.006	
*Tannerella forsythia*	BL	4.64 ± 1.78	4.72 ± 1.98	0.762
	4W	3.66 ± 1.86	1.81 ± 1.21 **	0.043
	8W	3.68 ± 2.05	2.29 ± 1.55 **	0.122
	*p*-value	0.067	0.002	
*Treponema denticola*	BL	3.06 ± 1.85	2.82 ± 1.74	0.7623
	4W	2.98 ± 1.60	1.26 ± 0.59 *	0.006
	8W	2.83 ± 1.66	2.01 ± 1.62	0.274
	*p*-value	0.641	0.050	

^1^*p*-value between test and control; ^2^*p*-value within test or control. ** *p* < 0.05 in comparison with baseline; * *p* < 0.1 in comparison with baseline. Undetermined bacteria were treated as 1 (log 10). *p*-value was determined by the Mann–Whitney U test between groups and by the Friedman test followed by the Wilcoxon signed-rank test with a Bonferroni correction for intragroup comparisons.

## Supplementary Materials

The following are available online at https://www.mdpi.com/2304-6767/8/4/123/s1, Figure S1: The relative abundance of genera with statistically significant differences between baseline and 4-weeks, and baseline and 8-weeks.

## Abbreviations

ANOVA	Analysis of variance
BH FDR	Benjamini and Hochberg false discovery rate
BOP	Bleeding on probing
CAL	Clinical attachment level
HOMD	Human oral microbiome database
OTUs	Operational taxonomic units
PCoA	Principal coordinates analysis
PCR	Plaque control record
PERMANOVA	Permutational multivariate analysis of variance
PPD	Probing pocket depth
QIIME	Quantitative insights into microbial ecology
SD	Standard deviation
SPT	Supportive periodontal therapy
TE buffer	Tris-EDTA (ethylene diamine tetra acetic acid) buffer
