# Supplementary figures and images for: Impact of Local Drug Delivery of Minocycline on the Subgingival Microbiota during Supportive Periodontal Therapy: A Randomized Controlled Pilot Study
Source: Dent J (Basel). 2020 Oct 27;8(4):123. doi: 10.3390/dj8040123 (PMC7711502; doi:10.3390/dj8040123)

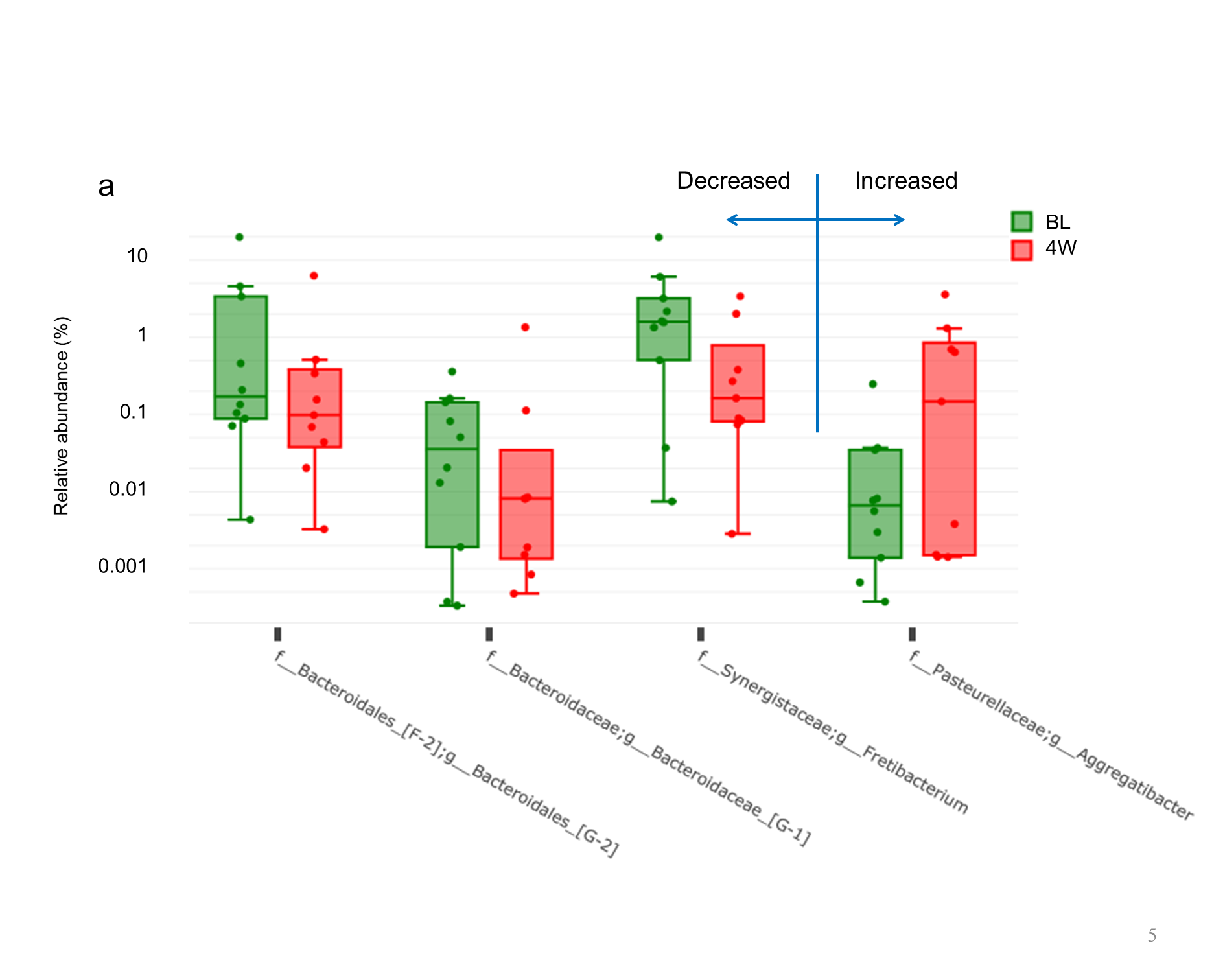

Supplement: Supplementary file 1 [file dentistry-08-00123-s001.zip › Figure S1_a.TIF]

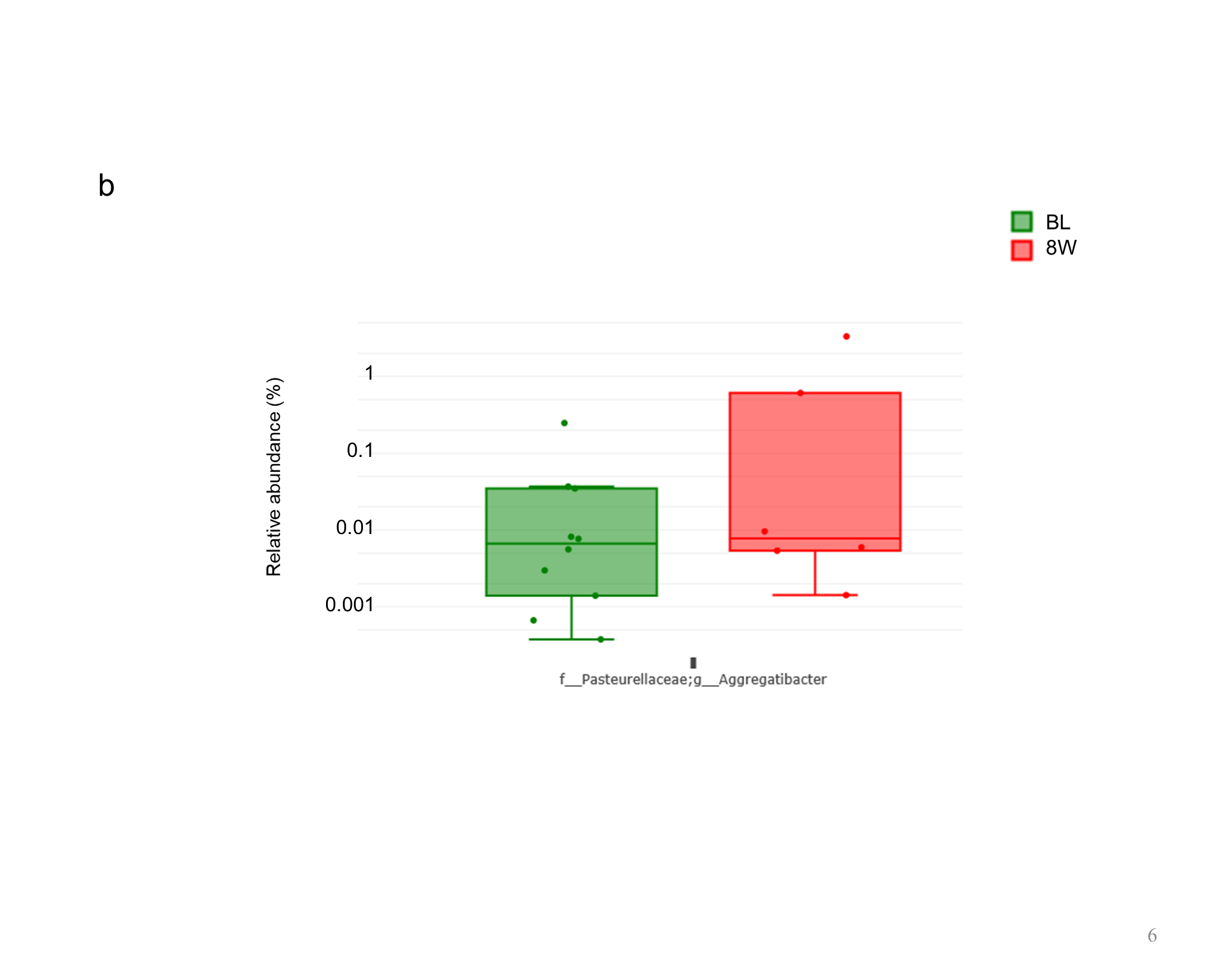

Supplement: Supplementary file 1 [file dentistry-08-00123-s001.zip › Figure S1_b.TIF]

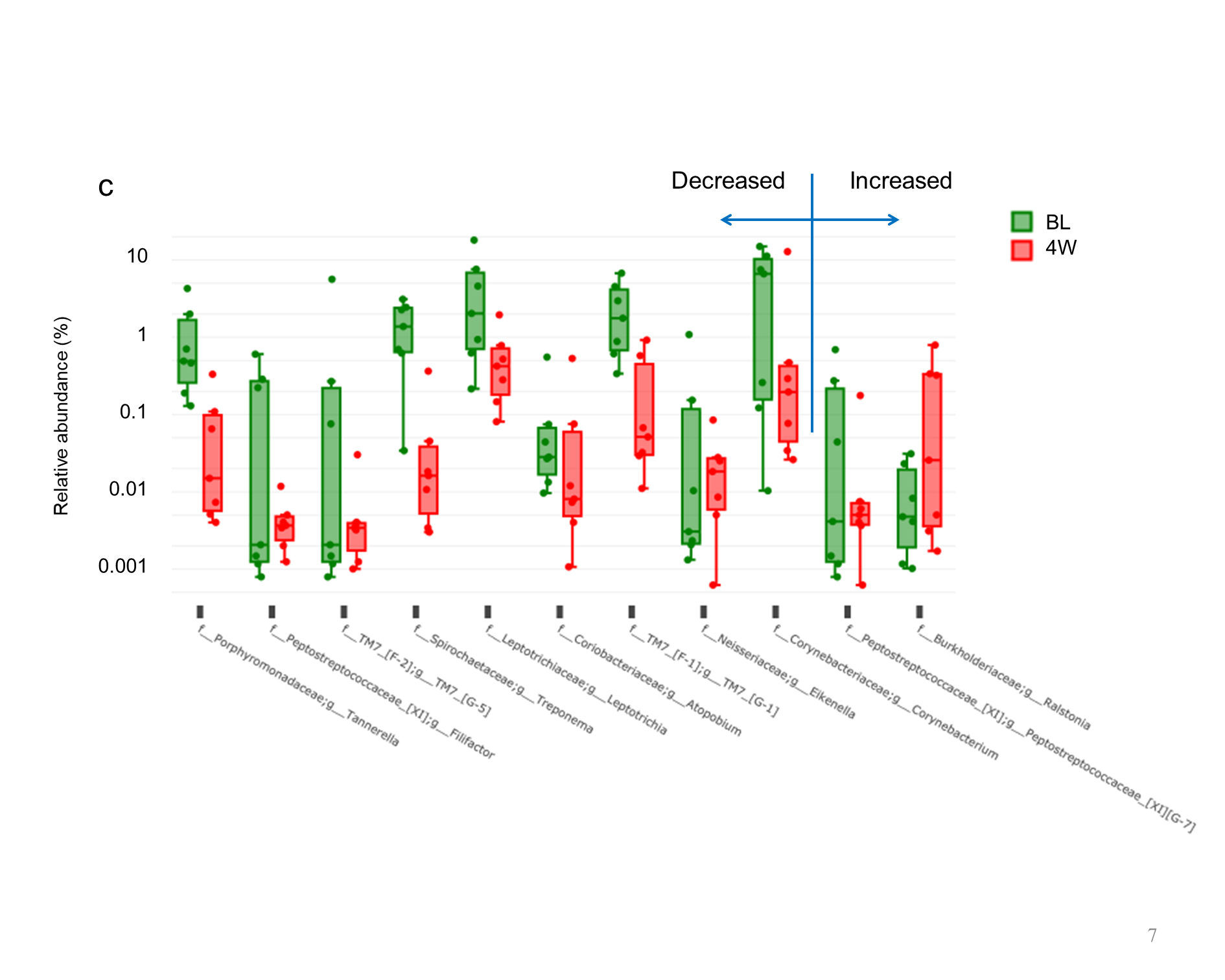

Supplement: Supplementary file 1 [file dentistry-08-00123-s001.zip › Figure S1_c.TIF]

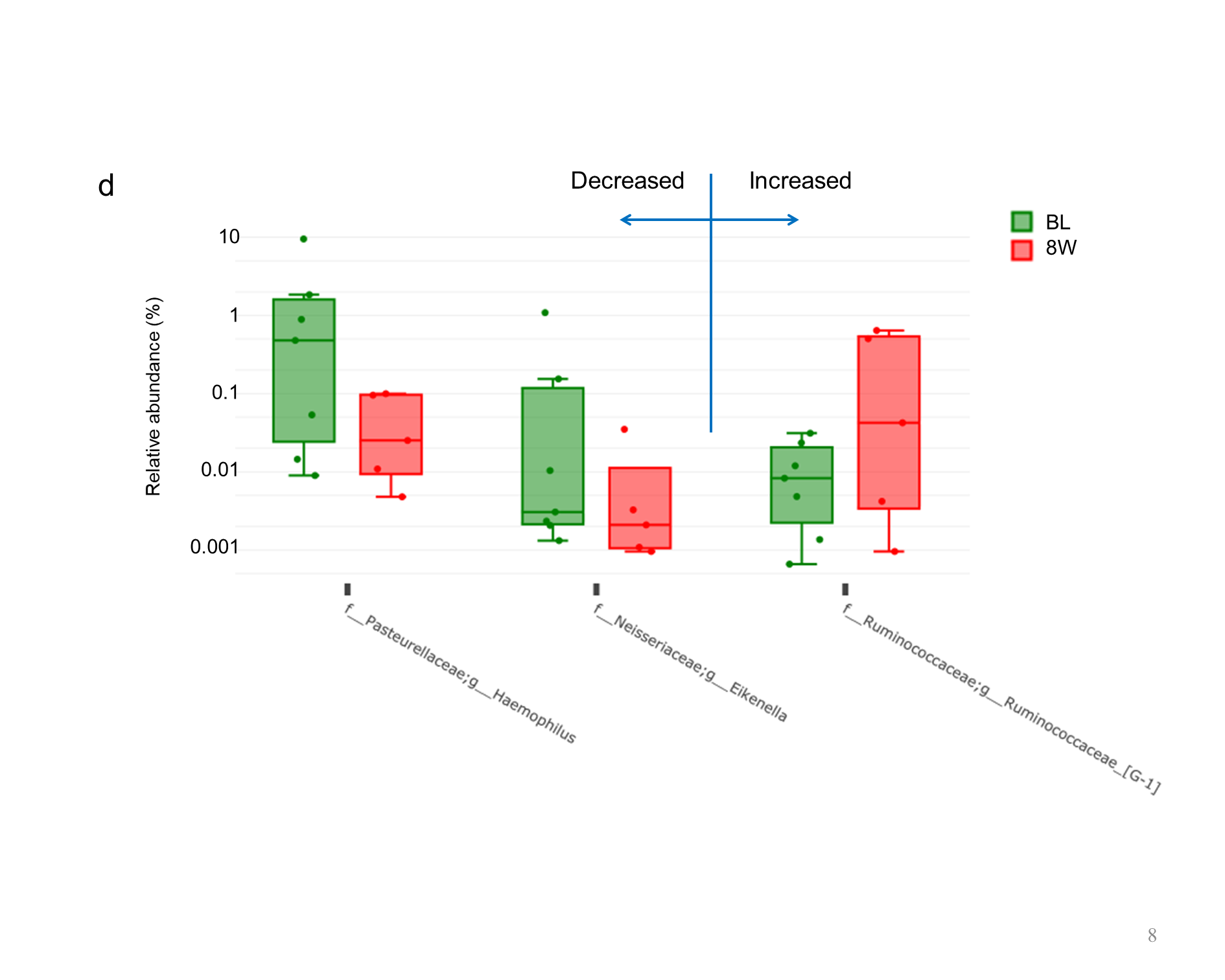

Supplement: Supplementary file 1 [file dentistry-08-00123-s001.zip › Figure S1_d.TIF]

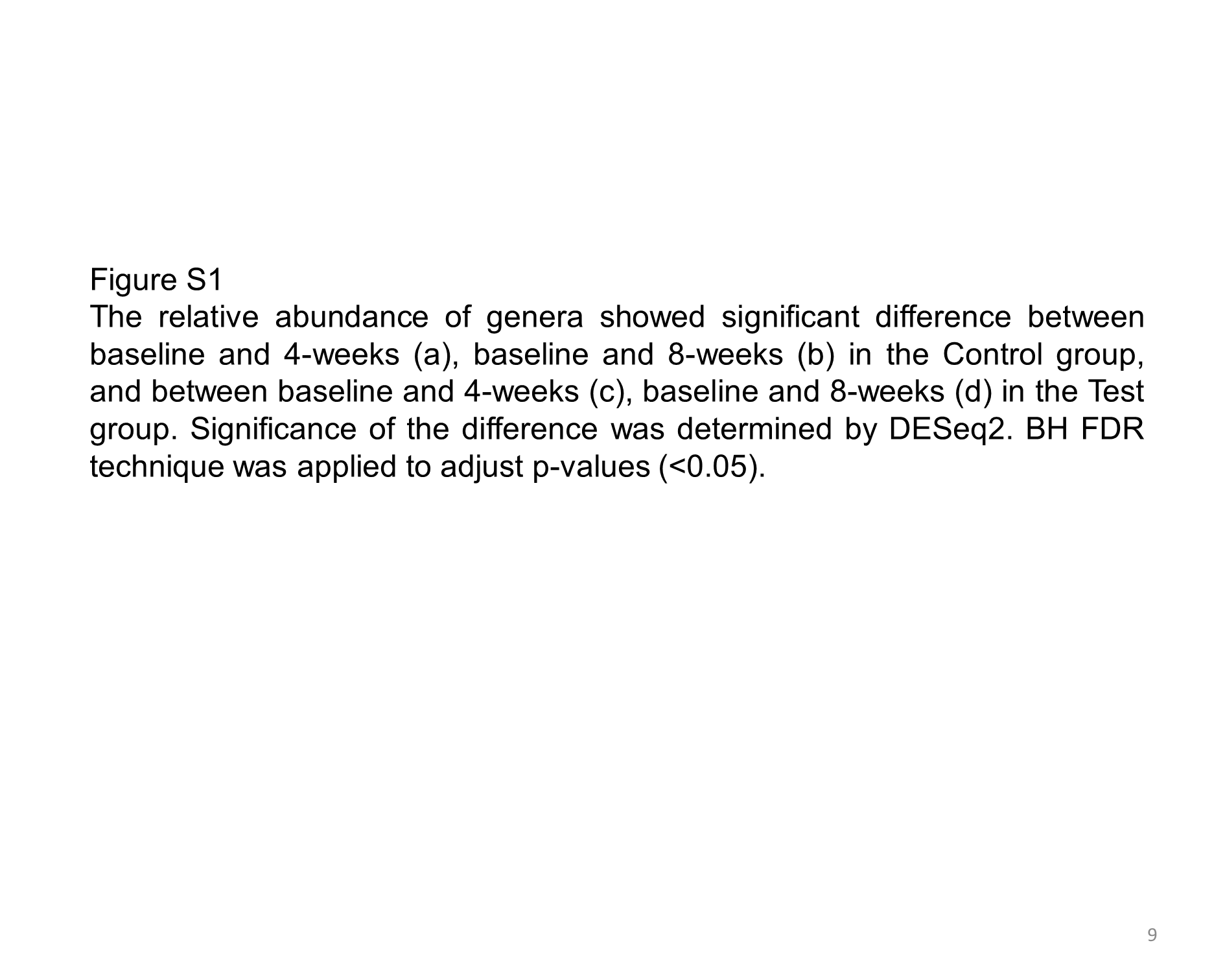

Supplement: Supplementary file 1 [file dentistry-08-00123-s001.zip › FiguresS1_legend.tif]
